# Missed a live match? Determinants of League of Legends Champions Korea highlights viewership

**DOI:** 10.3389/fpsyg.2023.1213600

**Published:** 2023-08-23

**Authors:** Yoonji Ryu, Hui Hwang, Jaehyun Jeong, Wonseok Jang, Gyemin Lee, Hyunwoong Pyun

**Affiliations:** ^1^College of Sport Science, Sungkyunkwan University, Suwon-si, Republic of Korea; ^2^Department of Smart ICT Convergence Engineering, Seoul National University of Science and Technology, Seoul, Republic of Korea

**Keywords:** esports, highlight viewership, League of Legends, League of Legends Champions Korea, outcome uncertainty, upset result, YouTube

## Abstract

This research aims to explore the determinants of the League of Legends Champions Korea (LCK) highlight views and comment counts. The data of 629 game highlight views and comment counts for seven tournaments were collected from YouTube. The highlight views and comment counts were regressed on a series of before-the-game factors (outcome uncertainty and game quality), after-the-game factors (sum and difference of kills, assists, multiple kills, and upset results), and match-related characteristics (game duration, evening game, and clip recentness). A multi-level least square dummy variable regression was conducted to test the model. Among the before-the-game factors, outcome uncertainty and game quality were significantly associated with highlight views and comment counts. This indicated that fans liked watching games with uncertain outcomes and those involving high-quality teams. Among the after-the-game factors, an upset result was a significant determinant of esports highlight views and comment counts. Thus, fans enjoy watching underdogs win. Finally, the sum of kills and assists only affected view counts, which indicated that fans prefer watching offensive games with more kills and a solo performance rather than teamwork.

## 1. Introduction

Esports is one of the fastest-growing sports industries and often shown as the form of professional sports league (Scelles et al., 2021). In 2020, the viewership of esports reached around 500 million, and the total estimated revenue of the esports industry was 160 billion US dollars (Newzoo, 2020). The biggest contributor to this rapid growth of esports has been its successful association with social live-streaming services (SLSS), particularly through platforms like Twitch and YouTube (Block and Haack, 2019). Unlike traditional professional sports, esports games are mostly broadcasted by SLSS, rather than traditional television (TV) channels.

SLSS viewers have different preferences than those of traditional TV. The most striking feature of SLSS is their interactive and synchronous nature. Every user can broadcast live videos, and viewers can interact with the broadcaster and other viewers via features like comments, chats, likes, and real-time donations (Scheibe, 2018). These unique features of SLSS seem to complement the interactive nature of video games; therefore, SLSS and esports have achieved remarkable success together (Bründl et al., 2022).

This emerging trend in esports has attracted scholarly attention. Existing research on esports and SLSS mostly focuses on the definition of esports (Wagner, 2006; Sjöblom and Hamari, 2017; Heere, 2018; Parry, 2019; Postma et al., 2022), motivations for watching esports (Brown et al., 2018a; Xiao, 2020), individual game streamers (Li et al., 2020; Xu et al., 2022), the relationship between playing video games and watching game streams (Jang and Byon, 2020; Jang et al., 2021), esports governance (Peng et al., 2020), esports players' wellbeing (Hong, 2022), and parasocial interaction (PSI) in SLSS (Leith, 2021; Wulf et al., 2021). Despite this initial scholarly interest, a few recent studies have tried to explain the esports industry (Newman et al., 2022), esports viewership (Watanabe et al., 2021), and esports replay viewership (Wang, 2022).

A highlights video is generally used as a teaser to attract new viewers to watch live streaming regularly (Bae and Kim, 2022). Video highlights are also popular in professional sports because fans cannot physically watch every game in real-time (McCammon, 2021). Compared to the monopolistic TV broadcasting services, SLSS provide consumers more freedom to specifically choose the video they want to watch. In addition, SLSS consumers are not restricted to a single TV for the entire family because they use their own mobile devices for streaming. Consequently, the demand for highlight videos has increased because consumers are faced with more choices in a limited time (Park et al., 2018).

The majority of esports fans are young men (Sjöblom and Hamari, 2017), namely “generation Z”, who are not as interested in watching live games as the older fans of traditional sports are (Silverman, 2020). Consequently, the consumption pattern of esports leagues is shifting from TV to mobile devices and from live games to highlights. Furthermore, the existence of various social media platforms enables sports organizations and teams to use short video clips, such as highlights, as a marketing tool to increase awareness and interest among existing and potential fans (Easton, 2020). Thus, the key to understanding esports leagues fans would be to identify the determinants of online highlights viewership, instead of the viewership of live games. In addition, studies on elite esports tournaments were limited yet (Scelles et al., 2021).

Herein, we aimed to explore the determinants of esports' highlight videos viewership. Considering the unique feature of SLSS, we identified the determinants of comment counts, which represents a new type of esports consumption via SLSS, as well as the traditional view counts. Focusing on the League of Legends Champions Korea (LCK), one of the most popular esports leagues in the world, this study successfully identified the determinants of highlights viewership and comment counts. The determinants, namely outcome uncertainty, the expected game quality, upset results, and evening games were found to be positively correlated to views and comment counts. In-game statistics, such as the sum of kills and assists, affected the view counts only; fans prefer offensive games with a solo performance. The results shed light on a deeper understanding of esports' fan demand and online viewership and have practical implications for esports and SLSS industries.

## 2. Literature review

### 2.1. Esports

Esports research has recently become a promising topic for sports, games, and communication scholars. In the early stage, most of esports research focused on the issue of whether esports should be included as sport or not (Heere, 2018; Parry, 2019; Postma et al., 2022). For example, Parry (2019) argued that esports do not qualify as sports based on the Olympic concept. However, Hamari and Sjöblom (2017) proposed an alternative perspective, suggesting that esports can be considered as a form of sports characterized by electronic systems facilitating the primary aspects of the sport. According to their definition, human-computer interfaces mediate the input of players and teams, as well as the output of the esports system. Despite the ongoing debate surrounding the classification of esports as a sport, it is undeniable that esports is situated within the sports industry and is a subject of interest in sport academic research (Hamari and Sjöblom, 2017; Heere, 2018).

Also, there are some studies related to esports governance (Peng et al., 2020) and esports player's related issues such as well-being and health (Bányai et al., 2019; Hong, 2022). Parry (2019) conducted an examination of the sustainability of the esports governance model, aiming to lay the groundwork for the creation of a more sustainable and balanced esports ecosystem. Their study focused on developing an ecosystem that considers the interests and rights of all stakeholders. Along with the esports governance literature, Hong (2022) conducted an investigation into the roles and responsibilities of esports stakeholders in safeguarding the health and well-being of esports players.

Extant research on esports consumers has mostly focused on the motivations of consuming esports. Some empirical studies have attempted to determine the differences and similarities in consumer behavior between esports and traditional sports (Lee and Schoenstedt, 2011; Brown et al., 2018b). For example, Brown et al. (2018b) aimed to delineate esports and traditional sports consumption and the contrast between them. More than 1,300 esports consumers answered surveys regarding the uses and gratifications obtained when consuming esports and mediated traditional sports. The results suggest that esports consumers seek media for both esports and traditional sports with similar motivations, specifically social support, fanship, and Schwabism, which is a form of information gathering intended to help one become more knowledgeable about sports (Ruihley and Hardin, 2011).

Some studies have focused on the phenomenon of esports streaming and streamers (Leith, 2021; Wulf et al., 2021). For instance, Wulf et al. (2021) were interested in the PSI among streamers of Twitch, one of the most popular video games streaming platforms. The results indicated that more interactive streams, where individual viewers were addressed by reacting to their chat messages, affected the PSI experiences positively.

Studies evaluating the relationship between playing the game and watching game streams, such as Jang and Byon (2020), found that those involved in recreational gameplay are more likely to consume esports media. Subsequently, Jang et al. (2021) classified esports media into two distinct categories: streamer's live streaming content and esports event broadcasts. They examined the mediating effect of live streaming esports content on the relationship between esports gameplay and esports event broadcast consumption. The results demonstrated that the intention to consume esports content through live streaming completely mediated the relationship between esports' recreational gameplay and event broadcast consumption.

To advance the understanding of esports consumers' behavior, several studies have analyzed the motivations and antecedents for esports media consumption (Sjöblom and Hamari, 2017; Xiao, 2020). Sjöblom and Hamari (2017) conducted an online survey of esports viewers from Reddit, Facebook, Twitter, and other game-related platforms. The results showed that knowledge acquisition was a positive predictor of esports viewership, suggesting that watching esports games helps in learning about the teams/players and other aspects of the games. Xiao (2020) also explored the factors that correlate with the behavioral intentions of watching esports based on the theory of reasoned action. The findings revealed that three behavioral belief-related factors (aesthetics, drama, and escapism) and subjective norms were positively associated with attitudes toward watching esports.

In general, earlier esports literature conducted surveys and interviews of esports fans to understand consumer behavior (Qian et al., 2020; Xiao, 2020). Recently, empirical studies have focused on analyzing factors affecting esports viewership using esports' game-level data are being focused on (Watanabe et al., 2021; Wang, 2022).

### 2.2. Determinants of sports fan demand

Identifying key determinants of esports fan demand would be important to systematically understand individuals' decision making for esports viewership. Most literature on the demand of sports fans focused on live attendance and identified before-the-game expectations as determinants because the decision to go to a game is made before the game starts. Several before-the-game factors have been identified, such as match quality, star player effect, and outcome uncertainty. Match quality based on team performance, such as league standing (Benz et al., 2009) and total league points, (Buraimo and Simmons, 2008; DeSchriver et al., 2016) particularly influence the fans' demand. In general, a better-performing team drives more attendance. Additionally, star players and players' salaries reportedly affect attendance demand in a positive way (Jewell, 2017; Sung and Mills, 2018; Humphreys and Johnson, 2020).

Fan preference toward outcome uncertainty has been widely studied. The uncertainty outcome hypothesis (UOH) of Rottenberg (1956) states that fans prefer uncertain outcomes compared to certain ones. Using betting odds as a proxy of win probability, early empirical evidence supported the UOH (Knowles et al., 1992; Rascher and Solmes, 2007; Benz et al., 2009). However, recent empirical studies have reported different results; fans prefer certain game outcomes, where either the home team wins or loses, compared to uncertain outcomes (Beckman et al., 2012; Martins and Cró, 2018; Sung and Mills, 2018; Besters et al., 2019). Coates et al. (2014) were the first to explain this contradictory evidence toward the UOH by applying the theoretical model of reference-dependent preference with a loss-averse agent. They explained that fans have expectations or references before going to a game, and the difference between their reference and the actual outcome generates additional (dis)utility. They also argued that fans prefer certain outcomes to uncertain ones because they do not want to have a large chance of getting disutility from an unexpected loss compared to the extra utility from an unexpected win (i.e., loss averse) when the outcome becomes more uncertain.

Competitive intensity is identified as a determinant of attendance in recent literature on European football leagues. As European football leagues have complicated prize structure depending on the final standing such as promotion and relegation, the European Champions League and the Europa League qualification, teams compete each other for more than one prize (Wagner et al., 2020). Several studies have attempted to measure the intensity of competitiveness according to different prizes in a league during ongoing season (Addesa and Bond, 2021; Hautbois et al., 2022).

Other match-related characteristics such as day of the week (Buraimo and Simmons, 2008), game time (Krumer, 2020), weather (Ge et al., 2020), and geographical distance between competing teams (Humphreys and Miceli, 2020; Sung and Pyun, 2023), are commonly used as determinants of sports demand. Home team market size and conditions, such as population, average income, stadium quality, and ticket prices, are the other identified determinants of attendance demand (Pyun et al., 2020).

Similar to studies on live attendance, most literature on TV demand have focused on before-the-game expectations, such as outcome uncertainty and superstar effects (Hausman and Leonard, 1997; Kanazawa and Funk, 2001; Forrest et al., 2005; Paul and Weinbach, 2007; Alavy et al., 2010; Tainsky, 2010). Allan and Roy (2008) and Cox (2018) explored the difference between live attendance and TV viewership. Empirical evidence suggests that the preferences of TV viewers are different from those of live attendance. Usually, live attendees are regarded as fans of the home team who strongly want their team to win (Humphreys and Zhou, 2015). However, TV viewership does not have this restriction; the viewer can be anyone who lives in the home (or away) team city, supports the home (or away) team, or lives in any region without a team preference, including international fans.Thus, there is a difference in the empirical evidence between live attendance and TV viewership, especially in their preference for outcome uncertainty (Feddersen and Rott, 2011; Cox, 2018). The results suggest that the fans' preferences may vary depending on the sports leagues or media platforms and thus, should be tested for esports fans via SLSS platforms.

### 2.3. Demand for highlights

While sports highlights or post-game shows have a long history in traditional TV services, only limited studies have focused on the viewership of highlights. Despite the limited number of studies, the existing literature can be categorized as follows: (i) studies focusing on the factors affecting viewership and (ii) studies analyzing the relationship between highlights viewership and TV viewership (Bae and Kim, 2022).

Dietl et al. (2003) assessed the determinants of highlights viewership of the German Bundesliga, and Salaga et al. (2022) studied the pre-game, actual game, and post-game viewership separately. Specifically, Salaga et al. (2022) categorized the determinants as follows: anticipated match characteristics (before-the-game expectation in our terms), temporal characteristics (match-related characteristics in our terms), substitutes and weather, and the actual match characteristics. Han et al. (2021) covered the viewership of highlight videos in the Korean soccer league and identified important determinants of online viewership, such as importance of the game, whether the match is a derby, in-game performance [actual match characteristics in Salaga et al. (2022)], and recentness of the highlight videos.

The demand for esports leagues remains unexplored in the academic field. A few recent studies have tried to explain the esports industry (Newman et al., 2022) and esports viewership (Watanabe et al., 2021). Most recently, Wang (2022) examined esports replay viewership data, focusing on the evidence supporting skill-based star effect and Butler and Butler (2023) tested the relationship between English Premier League highlight viewership and closed door games without attendance during the COVID-19 pandemic.

### 2.4. Present study

While live attendees and local TV viewers are often regarded as home team fans (Coates et al., 2014; Salaga et al., 2022), highlight viewers via YouTube can be anyone; they may be a fan of the home or away team, or a neutral fan. In such case, individual team-level measures cannot be applied, and team-level data are aggregated as game-level data for all variables.

Using a similar approach as that of Han et al. (2021) and Salaga et al. (2022), and given that highlights are generated after the live game, the highlights video demand factors can be broadly divided into three categories: before-the-game expectations, match-related characteristics, and after-the-game factors.

The factors determined before the game include match quality, super-star effects, and outcome uncertainty. For game quality, current team performance (e.g., league standing) within a season before the game starts is commonly used in the previous studies (Buraimo and Simmons, 2008; Benz et al., 2009; DeSchriver et al., 2016). However, current team performance measures are renewed at the beginning of every season, and team statistics early in the season are not reliable indicators as only a few games would have been played. Furthermore, winning against either a weaker or a stronger opponent is regarded as the same in these measures. To deal with these issues, we applied the Elo rating system as an indicator of game quality following Salaga et al. (2022). The Elo rating is a numerical system which covers all team performances while considering the quality of the opponents. Thus it outperforms other traditional measures, such as league standings or win percentages (Elo, 1978).

To capture the star players' effects, the number of all-star players or players' salary is often used in the previous literature (Humphreys and Johnson, 2020; Salaga et al., 2022). However, there is no all-star game in the LCK and the details of players' salaries are not open to public. Instead, LCK has two unique features compared to other sport leagues. (i) The reserve clause is not applied to any players, and every contract is one-year long. Thus, a player transfer during ongoing season is rare, and was not observed during the study period. (ii) Teams tend to use the same five players for every game during a season. By combining these two features, we argue that the team-year fixed effect captured the star players' effects.

We also identified the preference toward outcome uncertainty and game-related characteristics (day of the week and game time) as determinants of highlights viewership. However, other factors such as weather and home team market characteristics, were not included in our analysis. Even though LCK has a distinct home and away team for every game, the games are played in a neutral arena so that the distinction between home and away is not applicable.

Competitive intensity is not considered in this study either. Competitive intensity is often used in studies with European football leagues that have different prizes depending on league standings (e.g., international league qualification) (Wagner et al., 2020). LCK league has a relatively simple championship determination with playoff system.^1^ Also, as relatively large number of teams (five to six out of 10 teams) will make a playoff appearance, playoff contender could be most of teams during season.

Unlike studies on live attendance and TV viewership, highlights viewers decide to watch highlights after a game finished; therefore, factors determined after the match could affect viewership (Han et al., 2021; Salaga et al., 2022; Butler and Butler, 2023). For after-the-game factors, we include upset results, in-game statistics, and match duration.

In line with reference-dependent preference with loss aversion, unexpected game outcomes, especially unexpected losses, reportedly generate emotional cues that trigger the fans' subsequent behavior (Card and Dahl, 2011; Ge, 2018; Matti, 2021). In addition, deposition theory explains that enjoyment derived from watching a game depends on the emotional investment in the favorite team with the preferred game outcome (Raney, 2013). Therefore, to test the impact of unexpected outcomes on viewership, we included game outcomes and upset results in our analysis.

## 3. Empirical methods

### 3.1. Data

This study explores the highlights of the LCK league. The video game League of Legends (LoL) was released in 2009 by Riot Games and has become one of the most popular video game in the world. Based on this popularity, several professional leagues have been formed depending on geographical location. As of 2023, there are 9 professional leagues over 90 teams collectively. The LCK league is one of the four major LoL leagues in the world. Currently, ten teams participate in the LCK league, and each team plays double round-robin tournaments (18 rounds with 10 teams) as a regular season. Each game consists of three sets (best of three). The LCK league hosts two regular tournaments each year (spring and summer), and each tournament contains 90 games.

After the regular season games, the top five (until 2020 LCK Summer) to six teams advance to the playoff stage and follows a single elimination bracket system. The final championship is determined by the result of playoff stage. Until 2020 LCK Spring, the LCK utilized a relegation and promotion system referred to as the “LCK Promotion Tournament”. This system enabled teams from both the LCK and the secondary league, called Challengers Korea (CK), to contend for LCK spots. The bottom two teams in the LCK compete against the top two CK teams for LCK league appearance in the next tournament.

The LCK league runs a YouTube channel, and posts highlight videos after every game. We collected view and comment counts, of each game's highlight videos on the LCK's YouTube channel via the YouTube application programming interface (API). From the 2019 LCK Summer to the 2022 LCK Summer, we collected the viewership data on 629 game highlights from seven tournaments.^2^

For every game, we collected the duration of game time and the number of kills, assists, and multiple kills of both teams from https://lol.inven.co.kr. A kill is defined as a player killing the opponent player in the game, and is similar to scoring in traditional sports.^3^ Using the number of kills for each team, we calculated the sum of kills and the absolute value of the difference in kills to determine whether fans prefer offensive games (sum of kills) or games dominated by one team (difference in the kills). We also collected the number of assists and multiple kills as additional in-game statistics. Similar to traditional sports, an assist is defined as a player(s) helping teammates kill the opponent. Note that assists can be awarded to several players or there could be no assists for a kill. Thus, the sum of assists would indicate whether fans preferred teamwork or solo performances (more assists mean more teamwork). Multiple kills is defined as a player consecutively killing opponents within ten seconds. Multiple kills is usually an outcome of the in-game fighting which most players participate in. It is the most remarkable moment for fans, and the game's outcome is often determined during this fight.

We collected the betting odds for each game from https://www.oddsportal.com and used it as an indicator of game outcome uncertainty. Betting odds were converted to implied probabilities using Kuypers (2000)'s method to deal with the bookmakers' margins. Unlike traditional sports, every LCK game is played in a neutral arena, so a home-and-away distinction is not applicable.^4^ Thus, we used the squared difference between win probabilities for both teams as an uncertainty measure, following Buraimo and Simmons (2015); the smaller the difference, the greater the uncertainty. We also used the Theil measure, which has been commonly used as an indicator of outcome uncertainty (Beckman et al., 2012; Pawlowski and Anders, 2012; Serrano et al., 2015; Schreyer et al., 2018). Theil measure was calculated using the following formula:


(1) $$\begin{array}{l} \overset{2}{\underset{i=1}{\sum }}p_{i}log\left(\frac{1}{p_{i}}\right) \end{array}$$

where *p*_*i*_ is the win probability of team *i*. The higher the Theil measure, the greater is the uncertainty.

We calculated the Elo ratings for each team before every game. The 2018 LCK Summer tournament, two tournaments earlier than our sample, was set as a reference, and it was assumed that every team has the same quality (i.e., same Elo points). Subsequently, we calculated the Elo points for every game outcome the same method as that used by Ryall and Bedford (2010) and Nguyen et al. (2020). The sum of the Elo ratings of both teams were used to represent the overall game quality (Salaga et al., 2022).

We generated a binary “upset results” variable which was equal to one where a weaker team with poor betting odds won and zero otherwise. We also generated the interaction term between the upset dummy and absolute difference in win probability between the two teams; the impact of the upset results was expected to be higher when the upset is more unpredictable (i.e., the absolute difference in win probability was high).

Match-related characteristics, such as the day of the week and start time (before or after 6 pm), the interval between the posted date and data collected date to determine the recentness of the highlights clip, were collected.

Table 1 presents the summary statistics of the variables. The average view count was 127,234 and the average comment count was 237.5. The squared win difference was 0.23 and the sum of the Elo ratings was 3,015 on average. The average sum of the kills was 55.2, ranging from 22 to 114. The average difference in kills was 15.1. A total of 176 outcomes (27.9%) were upset results. The average duration of a game was 163 min and 49.4% of the games were played after 6 pm.

**Table 1:** Summary statistics.

	**Mean**	**SD**	**Min**	**Max**
View count	127,234	88,737	24,562	717,819
Comment count	238	252	18	1,499
Sq. win difference	0.231	0.2	0	0.736
Theil	0.568	0.114	0.257	0.693
Sum of Elo ratings	3015	95.2	2715	3318
Sum of kills	55.3	16.7	22	114
Diff. of kills	15.1	8.47	0	42
Sum of assists	129	39.9	49	280
Diff. of assists	38.3	21.7	0	122
Sum of multiple kills	7.83	3.63	0	22
Diff. of multiple kills	3.83	2.53	0	13
Upset	0.28	0.449	0	1
Match duration	163	36.5	85	255
Evening match	0.494	0.5	0	1
Clip age (days)	774	364	213	1,379
Observations	629			

Sq., Squared; Prob., probability; Diff., difference.

### 3.2. Analysis

To explore the determinants of the LCK league highlights view counts, the following empirical model was formulated:


(2) $$\begin{array}{l} ln{\left(viewcount\right)}_{ijwt}=\beta_{0}+X^{\prime }\gamma +Z^{\prime }\rho +W^{\prime }\theta +\alpha_{i}+\delta_{j}+\lambda_{t}+\epsilon_{ijwt} \end{array}$$

where *ln*(*viewcount*)_*ijwt*_ is the view count of the LCK league game for the home team *i* and away team *j*, in the week *w*, in the tournament *t*. *X* is a vector of variables that captures before-the-game expectations from the fans' perspective, including outcome uncertainty and overall game quality. For outcome uncertainty, we used the squared difference in the win probability and the Theil measure, separately. For overall game quality, we use the sum of the Elo ratings and the sum of the current league standing, separately.

*Z* is a vector of variables that represents in-game performance since the highlight videos are usually available after the game, and viewers may be aware of the game outcome before watching it. This vector includes the sum of kills and absolute value of the difference in kills to determine whether fans prefer offensive (more kills) and one-sided games. We further assessed whether other performance statistics (assists and multiple kills) could affect the view counts. *Z* also includes the upset results variable to identify whether fans prefer watching matches with upset outcomes. We also used the interaction between the upset dummy and absolute difference in win probability to determine whether the impact is higher when a upset was more unpredictable.

*W* is a vector of match-related characteristics, which include match duration, evening games, recentness of the highlights, day of the week fixed effects, and number of weeks within the tournament fixed effects.

α_*i*_ and δ_*j*_ are the home team *i* and away team *j* fixed effects, λ_*t*_ captures the tournament fixed effects. ϵ_*ijwt*_ is a heteroscedastic unobservable error term. The equation error term was assumed to be correlated within the home team *i*, and we clustered the standard errors accordingly. We performed multi-level (*i*, *j*, and the tournament) least square dummy variable (LSDV) regression.

As discussed earlier, the stars' effects were not well addressed in Equation (2) because relevant data (e.g., players' salary) was not observable. To address this issue, we performed the same multi-level LSDV regressions with home team-year and away team-year fixed effects. Both team-year fixed effects would capture the team and year specific variations, such as a fixed roster within a year for every LCK team.

On YouTube, fans can write a comment during or after watching a highlights video. To explore the determinants of the comment counts of the highlight videos, the following empirical model was formulated:


(3) $$\begin{array}{l} ln{\left(commentcount\right)}_{ijwt}=\beta_{0}+X^{\prime }\gamma +Z^{\prime }\rho +W^{\prime }\theta +\alpha_{i}+\delta_{j}+\lambda_{t}+\epsilon_{ijwt} \end{array}$$

where *ln*(*commentcount*)_*ijwt*_ is the comment count of the LCK league game video of the home team *i* and away team *j*, in the week *w*, in the tournament *t*. Every other empirical setting was the same as that in Equation (2).

## 4. Results

Table 2 presents the main results of the logged view count from Equation (2). Column (1) presents the results of home team and away team fixed effects models, and Columns (2) and (3) include the results of the home team-year and away team-year fixed effects models. The estimated coefficients on the squared win probability difference were negative but not significant in Model (1) and (2), and significantly negative in Model (3). The negative coefficient indicates that LCK fans prefer unpredictable games as predicted by the UOH.

**Table 2:** Determinants of view counts.

	**(1)**	**(2)**	**(3)**
**Dependent variable: logged view counts**			
Sq. win difference	−0.018	−0.080	−0.147**
	(0.060)	(0.061)	(0.062)
Sum of Elo ratings	0.002***	0.001***	0.001***
	(0.000)	(0.000)	(0.000)
Sum of kills	0.002**	0.002*	0.002*
	(0.001)	(0.001)	(0.001)
Diff. of kills	−0.001	−0.001	−0.001
	(0.001)	(0.001)	(0.001)
Upset	0.072**	0.082**	
	(0.032)	(0.031)	
Upset × Diff. win prob.			0.245***
			(0.068)
Match duration	0.000	0.000	0.000
	(0.000)	(0.000)	(0.000)
Evening match	0.165***	0.161***	0.163***
	(0.019)	(0.019)	(0.019)
Clip age	0.008*	0.008*	0.008*
	(0.004)	(0.004)	(0.004)
Home team fixed effects	Yes	No	No
Away team fixed effects	Yes	No	No
Home team-year fixed effects	No	Yes	Yes
Away team-year fixed effects	No	Yes	Yes
Tournament fixed effects	Yes	Yes	Yes
R-squared	0.829	0.862	0.864
N	629	629	629

* *P* < 0.1;

** *P* < 0.05;

*** *P* < 0.01.

Cluster-corrected standard errors at home team level in parentheses.

Sq., Squared; Prob., probability; Diff., difference.

The estimated coefficients on the sum of the Elo ratings were positive and significant consistently, indicating that LCK fans prefer better quality games. The sum of kills was a significant predictor of view count. An additional kill increased the view count by 0.2%. The results indicate that fans prefer offensive games with more kills. However, the absolute value of the difference in kills had no significant impact on the highlights viewership, indicating that fans had no preference for one-sided games.

The estimated coefficients on upset results were positive and statistically significant; 8.2% more fans watched the highlights video when the game had an upset result. In addition, the interaction term between an upset and the absolute difference in win probability was positive and statistically significant. This suggests that more fans watch the highlights of upset results when the upset is more unpredictable.

The game duration did not affect the highlight view counts significantly. Evening games drove significantly more view counts, and around 16% more views were recorded for evening games. This may indicate that the LCK league arranges more popular games in the evening. Additionally, older videos have more view counts, as expected.

Using Model (3) in Table 2 as a main specification, we further assessed whether our results were sensitive with different measures. Table 3 presents the logged view count with home team-year and away team-year fixed effects. Column (1) shows our main results, Model (3) in Table 2, as a reference. The column (2) includes the Theil and sum of the current league standings as alternative measures for outcome uncertainty and game quality, respectively. Similar results were reported compared in Columns (1) and (2). The estimated coefficient on Theil was positive and significant, indicating that fans prefer unpredictable games. Additionally, the estimated coefficient on the sum of the current league standings was negative and significant, indicating that fans prefer high quality games with better ranked teams.

**Table 3:** Determinants of view counts with alternative measures.

	**(1)**	**(2)**	**(3)**	**(4)**	**(5)**
**Dependent variable: logged view counts**					
Sq. win difference	−0.147**		−0.143**	−0.140**	−0.170**
	(0.062)		(0.061)	(0.059)	(0.059)
Theil		0.267**			
		(0.100)			
Sum of Elo ratings	0.001***		0.001***	0.001***	0.001***
	(0.000)		(0.000)	(0.000)	(0.000)
Sum of standings		−0.019***			
		(0.005)			
Sum of kills	0.002*	0.002			0.007***
	(0.001)	(0.001)			(0.002)
Diff. of kills	−0.001	−0.002			−0.004
	(0.001)	(0.001)			(0.004)
Sum of assists			0.000		−0.002***
			(0.000)		(0.001)
Diff. of assists			−0.001		0.001
			(0.000)		(0.001)
Sum of multiple kills				0.003	−0.001
				(0.003)	(0.003)
Diff. of multiple kills				−0.003	−0.000
				(0.005)	(0.005)
Upset × Diff. win prob.	0.245***	0.246***	0.247***	0.247***	0.253***
	(0.068)	(0.067)	(0.068)	(0.069)	(0.067)
Match duration	0.000	0.000	0.001	0.001***	0.000
	(0.000)	(0.000)	(0.000)	(0.000)	(0.000)
Evening match	0.163***	0.158***	0.163***	0.164***	0.164***
	(0.019)	(0.018)	(0.018)	(0.019)	(0.020)
Clip age	0.008*	0.008	0.008*	0.008	0.007
	(0.004)	(0.005)	(0.004)	(0.004)	(0.004)
Home Team-year fixed effects	Yes	Yes	Yes	Yes	Yes
Away Team-year fixed effects	Yes	Yes	Yes	Yes	Yes
Tournament fixed effects	Yes	Yes	Yes	Yes	Yes
R-squared	0.864	0.862	0.862	0.862	0.865
N	629	629	629	629	629

*Note:*

* *P* < 0.1;

** *P* < 0.05;

*** *P* < 0.01.

Cluster-corrected standard errors at home team level in parentheses.

Sq., Squared; Prob., probability; Diff., difference.

In Columns (3) to (5), we tested the impact of various in-game statistics on view counts. Column (3) includes the sum and difference of assists, instead of kills. While estimated coefficients on other variables remained unchanged, assists appear to have no effect on view counts. Column (4) includes the multiple kill counts as in-game performance statistics; it did not significantly affect view counts. Column (5) includes every in-game statistics in the model; kills, assists, and multiple kills. This model suggests that fans prefer more kills; one more kill increased the view count by 0.7%. Furthermore, fans prefer less assists; one additional assist would reduce the view counts by 0.2%. Multiple kills did not change the view counts.

Finally, we identified the determinants of comment counts in LCK match highlight videos. Table 4 reports the results of the logged comment counts from Equation (3). Every model applies home team-year and away team-year fixed effects.

**Table 4:** Determinants of comments counts.

	**(1)**	**(2)**	**(3)**
**Dependent variable: logged comment counts**			
Sq. win difference	−0.428***		−0.447***
	(0.086)		(0.101)
Theil		0.760***	
		(0.153)	
Sum of Elo ratings	0.002***		0.002***
	(0.000)		(0.000)
Sum of standings		−0.022***	
		(0.007)	
Sum of kills	0.001	0.001	0.004
	(0.002)	(0.001)	(0.003)
Diff. of kills	0.001	0.000	0.005
	(0.002)	(0.002)	(0.004)
Sum of assists			−0.002
			(0.001)
Diff. of assists			−0.001
			(0.002)
Sum of multiple kills			0.009
			(0.007)
Diff. of multiple kills			−0.009
			(0.008)
Upset × Diff. win prob.	1.091***	1.094***	1.107***
	(0.099)	(0.101)	(0.096)
Match duration	−0.000	−0.000	−0.000
	(0.001)	(0.001)	(0.001)
Evening match	0.266***	0.263***	0.269***
	(0.035)	(0.034)	(0.036)
Clip age	0.006	0.006	0.004
	(0.009)	(0.009)	(0.009)
Home team-year fixed effects	Yes	Yes	Yes
Away team-year fixed effects	Yes	Yes	Yes
Tournament fixed effects	Yes	Yes	Yes
R-squared	0.804	0.800	0.805
N	629	629	629

^*^p < 0.1;

^**^p < 0.05;

*** p < 0.01.

Cluster-corrected standard errors at home team level in parentheses.

Sq., squared; Prob., probability; Diff., difference.

Results revealed similar fan preferences regarding outcome uncertainty and game quality for comment counts than for view counts. Estimated coefficients on squared win difference and Theil measure were significantly negative and positive, respectively. Fans preferred to write a comment when the game outcome was more uncertain. Parameter estimates on the Elo ratings (or current league standings) were positive (or negative) and statistically significant; fans posted more comments for high quality games. Estimated coefficients on the interaction between the upset dummy and absolute difference in win probability were positive and statistically significant. Upset results generated more attention from fans than usual outcome did, resulting in more posted comments. More comments are posted for evening games (26%). In-game statistics, such as kills, assists, multiple kills, match duration, and clip age did not affect the comment counts.

## 5. Discussion

This study attempted to identify the determinants of the LCK league highlight views and comment counts. Using the data from the LCK channel on YouTube, we discovered that outcome uncertainty, game quality, sum of kills and assists, upset results, and evening matches were associated with view counts. For comment counts, outcome uncertainty, game quality, upset results, and evening matches were identified as determinants.

Regarding outcome uncertainty, both the squared win probability difference and Theil measure were significantly associated with the view and comment counts. The results support the UOH for highlight viewers, which was consistent with those of previous studies on TV viewership for traditional sports (Paul and Weinbach, 2007; Tainsky, 2010; Cox, 2018).^5^ These results are consistent with those of a study on Korean soccer league live attendees (Sung and Pyun, 2023).

The game quality influences the highlight views and comment counts. This indicates that fans prefer watching games involving a team with higher Elo ratings or lower league standings (i.e., a better team). This result is consistent with those of previous findings on live attendees (Buraimo and Simmons, 2008; Benz et al., 2009; DeSchriver et al., 2016), TV viewership (Kim et al., 2021; Wills et al., 2022), and esports replays (Wang, 2022).

The sum of the kills was a strong predictor of highlight views. LCK fans, or at least highlight viewers prefer offensive games involving more kills than defensive games. This result is consistent with that of the previous findings of a positive association between in-game performance and game attendance (Han et al., 2021; Johnson, 2021). The sum of kills is comparable to the scores in traditional sporting games. Previous studies have demonstrated that sports fans prefer to watch exciting games with high scores than boring games with low scores (Paul and Weinbach, 2007; Alavy et al., 2010). Because one of the key motivations of playing and consuming esports media is hedonic motivation, the sum of kills which may increase viewers' arousal and enjoyment level, predicts the highlight views (Jang and Byon, 2020). The sum of assists was negatively associated with view counts, indicating that fans prefer to watch solo performances, not teamwork. This may indicate that fans want to acquire skills by watching gameplays, mostly solo performances (Sjöblom and Hamari, 2017). On the other hand, the results may reveal the nature of highlight videos. A highlight video of an offensive game with many kills contains more fighting moments that highlight viewers want to watch.

Upset results have a strong impact on highlight views, and the impact increases when the upset results are unexpected. This indicates that fans prefer to watch highlight clips and post comments when the underdogs defeat the favorites. This result is consistent with the one in Butler and Butler (2023), and supports the arguments presented by Card and Dahl (2011), Ge (2018), and Matti (2021) that an unexpected outcome (i.e., a game with upset result) can activate an emotional cue and influence the subsequent actions of the fans. In our context, the unexpected wins and losses might have generated emotional cues, triggering the subsequent behavior of watching highlights and posting comments.

Deposition theory suggests that enjoyment from watching a game depends on an emotional investment in a favorite team with the preferred game outcome (Raney, 2013). This may suggest that highlight viewers are more likely to be fans of the winning team. This could be attributed to the fact that the decision to watch highlight videos is made after the game ends when the fans already know the results. Thus, fans would watch a highlights or post a comment more if the game result is their preferred outcome with more kills. Moreover, an upset win may drive a huge enjoyment to fans.

Although outcome uncertainty, game quality, and upset results affect the highlight views and comment counts in a similar manner, in-game performance does not alter comment counts. Fan behaviors on social media like YouTube can be categorized as consuming, contributing, and creating (Muntinga et al., 2011). Among these behaviors, consuming generates the lowest involvement such as watching a clip. Contributing generates mid-level involvement, such as generating an interaction between users, including posting comments (Kim and Yang, 2017).^6^ Using a similar framework, Buzeta et al. (2020) reported different impacts of motivation with uses and gratifications theory on user behaviors in broadcasting social media (e.g., YouTube) (Muntinga et al., 2011). Motivating factors, such as empowerment and remuneration, affect the consumption (watching a clip in our study) and contribution (posting a comment in our study) of media in a similar way. Other motivating factors, including entertainment, integration and social interaction, and information only affect the consumption, but not the contribution, of media content. Our results indicate that before-the-game expectations and upset results trigger remuneration motivations (i.e., the fans' desire), thus affecting view and comment counts in the same way. However, in-game performances may trigger information motivations (i.e., detailed game information). Thus, only view counts were affected by these factors.

## 6. Conclusion

We aimed to understand esports viewers' consumer behavior by exploring the determinants of LCK highlight views and comments. First, among the before-game expectations, outcome uncertainty and the game quality were significantly associated with view and comment counts. This indicates that fans prefer watching unpredictable games and those with high quality teams. Second, the upset results were significant predictors of esports' highlight views and comment counts. Thus, fans enjoy watching the underdogs win. Finally, in-game statistics only affect the view counts; fans prefer watching offensive games with more kills, and a solo performance rather than teamwork.

Using fan demand for highlight video clips, we examined their preferences on in-game performance. This was not adequately evaluated in existing literature because game viewers (either live attendees or TV viewers) do not know these factors before watching the game. Although we only evaluated the impact of basic in-game statistics in the LoL game, several other factors, such as gained/spent gold, killed dragons, and total damages to the opponents are also available owing to the native digital nature of esports (Taylor, 2020). Thus, preference of esports in-game statistics requires further testing.

Like other studies on TV viewership (Cox, 2018) and esports replays (Wang, 2022), this study could not identify if each viewer was a fan of one team, fan of the other team, or had no favorites. Using full text comments and user identification, fans can be appropriately identified (Wang and Fan, 2022). As the before-the-game expectation, in-game performance, and game outcomes are applied in the opposite way depending on which team a fan supports (e.g., one team winning means the opponent losing), fan identification may be the key to understanding SLSS viewership in future research.

The findings of this study can act as guidelines for the highlight clips producers. Recently, the demand for highlights has increased among sports fans, with several sports leagues such as the National Basketball Association, National Hockey League, and Korean Baseball Organization providing highlights using artificial intelligence (AI) to meet the high demands. AI highlights are technology-based automated highlight videos generated without a human editor, for the sole purpose of generating a large number of clips in a short time. In addition to developing AI algorithms, the study results can be used for generating customized highlights for esports fans.

Further research is required to obtain more practical implications. Bae and Kim (2022) demonstrated that highlights viewership often leads to an increased live game viewership. Furthermore, the LCK league may be able to attract new fans using highlight videos. This relationship can be tested via matching live game viewership data to that of highlight view counts. Although YouTube is one of the most popular SLSS in Korea, there are several other similar services such as Twitch and Afreeca TV. The quality of the highlights video also affects watching decisions. However, this was not deliberated on in this study. Moreover, there are potential variables, such as star player effects, that might affect esports viewers. We could not include them in the model due to the limitation of data accessibility. Future research should consider more factors that may impact esports demands. Finally, since we only focused on a single esports, LoL, future research should examine determinants in other esports such as Dota2 or Overwatch.

## Data availability statement

Publicly available datasets were analyzed in this study. The raw data supporting the conclusions of this article will be made available by the authors, without undue reservation.

## Author contributions

YR wrote the manuscript draft and performed the initial data analysis. HH and JJ collected and organized the data and assisted with statistical analyses. WJ and GL contributed to the conception and design of the study and assisted with editing the manuscript. HP provided supervision over the project, analyzed the data, and edited the manuscript as a corresponding author. All authors contributed to the article and approved the submitted version.

## Footnotes

^1^International league qualification is determined by the final standing from the result of playoff games. This study only focuses on regular season games, not playoff games.

^2^One game was canceled due to the COVID-19 pandemic.

^3^Note that a win is not determined by the kill difference. A team will win when it conquers the opponent's base.

^4^LCK league assigns home and away team for every game for constructing double round robin system though.

^5^Note that previous studies on the England Premier League TV viewership have reported no evidence for supporting UOH (Buraimo and Simmons, 2015; Scelles, 2017).

^6^Creating produces the highest involvement, such as sharing a clip, which was not observed in this study.
